# Preclinical models for evaluating psychedelics in the treatment of major depressive disorder

**DOI:** 10.1111/bph.17370

**Published:** 2024-10-28

**Authors:** Laith Alexander, Dasha Anderson, Luke Baxter, Matthew Claydon, James Rucker, Emma SJ Robinson

**Affiliations:** 1 Department of Psychological Medicine, Institute of Psychiatry, Psychology and Neuroscience, King’s College London, UK; 2 South London and the Maudsley NHS Foundation Trust, London, UK; 3 School of Physiology, Pharmacology & Neuroscience, University of Bristol, UK

## Abstract

Psychedelic drugs have seen a resurgence in interest as a next generation of psychiatric medicines with potential as rapid-acting antidepressants (RAADs). Despite promising early clinical trials, the mechanisms which underlie the effects of psychedelics are poorly understood. For example, key questions such as whether antidepressant and psychedelic effects involve related or independent mechanisms are unresolved. Preclinical studies in relevant animal models are key to understanding the pharmacology of psychedelics and translating these findings to explain efficacy and safety in patients. Understanding the mechanisms of action associated with the behavioural effects of psychedelic drugs can also support the identification of novel drug targets and more effective treatments. Here we review the behavioural approaches currently used to quantify the psychedelic and antidepressant effects of psychedelic drugs. We discuss conceptual and methodological issues, the importance of using clinically relevant doses and the need to consider possible sex differences in preclinical psychedelic studies.

## Introduction

Treating major depressive disorder (MDD) remains a significant challenge, with current pharmacological therapies largely based on the serendipitous discovery of the antidepressant effects of drugs such as the tricyclic antidepressants and monoamine oxidase inhibitors in the 1950s. Second generation treatments were developed based on achieving similar pharmacological effects at receptors linked to their efficacy, but with more selective modulation leading to reduced side effects. However, these treatments are not effective in all patients and side effects remain an issue, therefore new and more effective treatments are needed. An estimated 30-40% of patients fail to respond to current treatments (Al-Harbi, 2012) and many experience challenging side effects including emotional blunting (Marazziti et al., 2019). Conventional antidepressant treatments have a delayed onset of action meaning the subjective clinical benefits typically do not emerge until several weeks after starting treatment (Frazer & Benmansour, 2002).

In 2000, Berman et al. reported a rapid and sustained antidepressant effect following a single low dose infusion of the N-methyl-D-aspartate (NMDA) antagonist, ketamine. The effects persisted for days, and even weeks in some patients, long after the drug had been metabolised and excreted. In recent clinical trials similar rapid and sustained antidepressant effects have been seen following single doses of the serotonergic psychedelics (referred to as psychedelics in the remainder of this article) psilocybin (Carhart-Harris et al., 2016; Goodwin et al., 2022; Von Rotz et al., 2023) and N,N-dimethyltryptamine (DMT) (D’Souza et al., 2022). These findings suggest that a novel class of rapid-acting antidepressants (RAADs) act through a mechanism distinct from conventional antidepressants. RAADs seem to be effective in treating patients where conventional antidepressants have failed to produce a response – so called ‘difficult to treat depression’ or ‘treatment-resistant depression’ (TRD). Whether these antidepressant effects will be similarly effective in patients with major depressive disorders who currently receive selective serotonin reuptake inhibitor (SSRI) as a first line treatment remains to be fully investigated but psilocybin has been granted FDA breakthrough status for both MDD and TRD, and can be prescribed for TRD in Australia. These drugs offer the potential to improve clinical outcomes for treatment-resistant patients and provide researchers with new avenues to explore in terms of identifying novel drug targets.

The suggestion that psychedelics may be beneficial in treating psychiatric disorders is not new. Throughout the 1950s and 1960s many studies were published indicating their clinical efficacy, particularly when used to facilitate psychological interventions (Nichols & Walter, 2021). From the mid-1960s, psychedelics increasingly became viewed as drugs of abuse and, following the Drug Amendments in 1962, further research effectively halted until very recently. As a result, much of the early research into their clinical efficacy pre-dates many recent advances in neuroscientific methodology and, consequently, knowledge about the underlying mechanisms of these compounds is limited. For example, it remains unknown whether the changes in perception and hallucinatory effects induced by psychedelics are necessary for clinical efficacy in MDD. If the mechanisms underlying the antidepressant effects of these drugs can be elucidated, then it may be possible to identify novel drug targets or better understand how to maximise the clinical benefits of these drugs while reducing their subjective effects. Animal behavioural studies play a crucial role in elucidating the mechanisms underlying the clinical effects of psychedelics. This review considers the animal studies which have been used to understand their pharmacology and future strategies for a new generation of psychiatric medications.

### Psychedelic drug pharmacology

**Figure 1:**
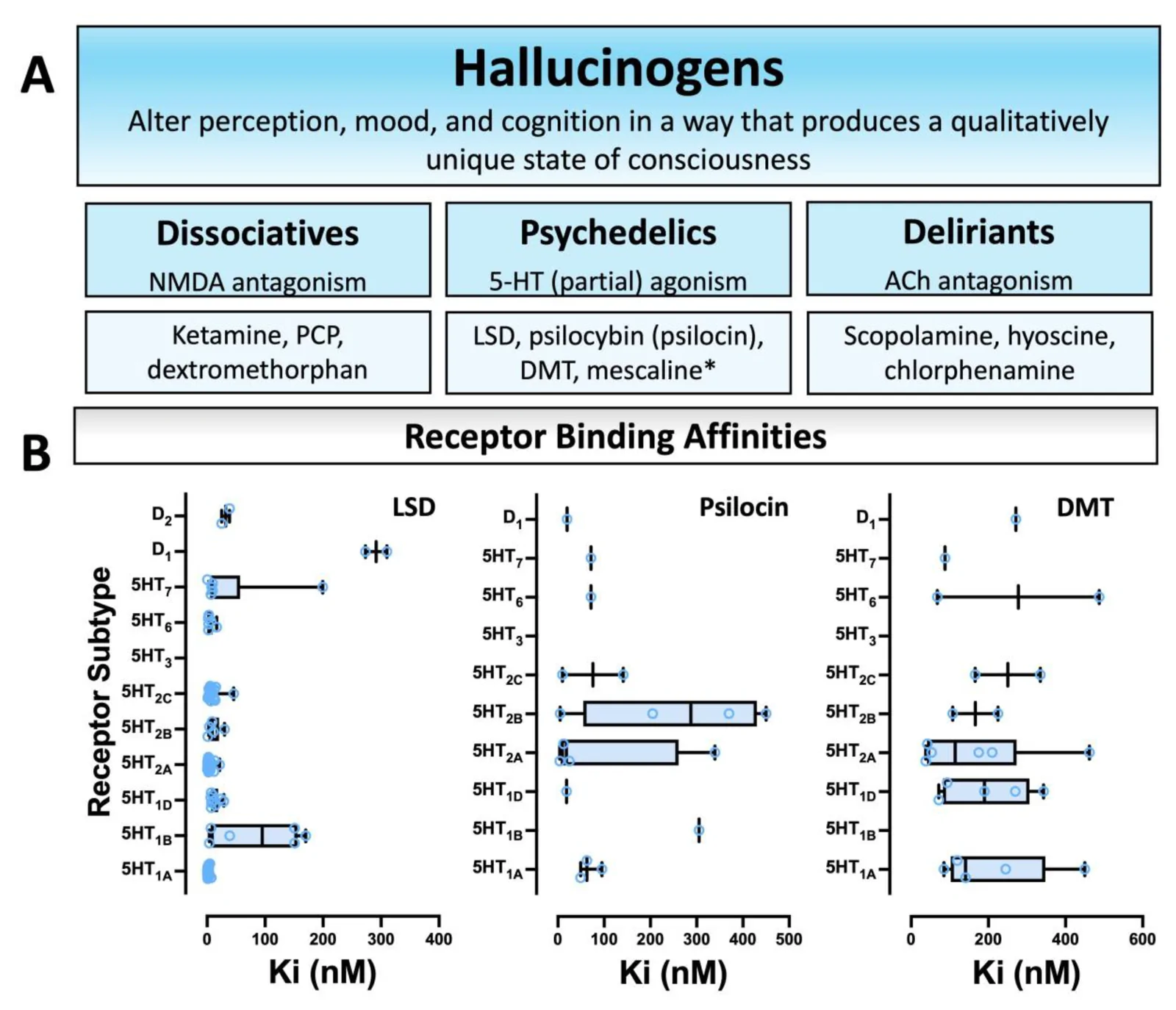
**A)** Psychedelics are a type of hallucinogen, with distinct subjective effects compared to deliriants e.g. scopolamine, and dissociatives e.g. ketamine. **B)** Psychedelic drugs and their affinity for serotonin and dopamine receptors. Data obtained from PDSP database: https://pdsp.unc.edu/databases/kidb.php (Accessed: January 10, 2023). *Mescaline is another a prototypical psychedelic, however will not be discussed further in this review due to a lack of animal studies for this drug. 5-HT serotonin; NMDA, *N*-methyl-*D*-aspartate; ACh, acetylcholine; DMT, N,N-dimethyltryptamine; LSD, lysergic acid diethylamide; DOI, 2,5-Dimethoxy-4-iodoamphetamine; PCP, phencyclidine.

Hallucinogens are broadly divided into three categories based on their pharmacology and the subjective experience they induce (Figure 1A). Psychedelics are a class of hallucinogenic drug which induce an altered state of consciousness dominated by changes in sensory perception. Psychedelic drugs broadly fall into three categories based on their molecular structures: tryptamines (including psilocybin and its primary active metabolite psilocin, DMT, 5-Methoxy-N,N-dimethyltryptamine (5-MeO-DMT), phenethylamines/phenylalkylamines (including mescaline, 2,5-Dimethoxy-4-bromophenethylamine [2C-B], 2-(4-Iodo-2,5-dimethoxyphenyl)-N-[(2-methoxyphenyl)methyl]ethanamine [25I-NBOMe], and 1-(4-Iodo-2,5-dimethoxyphenyl)propan-2-amine [commonly known as 2,5-Dimethoxy-4-iodoamphetamine, DOI], and lysergamides/ergolines (including lysergic acid diethylamide or lysergide [LSD]). Despite the variability in their chemical structures, the unifying feature of these psychedelic compounds is their affinity for serotonin (5-Hydroxytryptophan, 5-HT) receptors within the central nervous system (CNS). The ‘classical’ psychedelics – psilocybin, DMT, and LSD – are serotonergic full or partial agonists with most of their effects attributed to activity at 5-HT_1A_ and 5-HT_2A_ receptors. As illustrated in Figure 1B, their relative affinities for the serotonin receptor subtypes differ and their activities at other receptors, such as dopamine and histamine receptors (Halberstadt & Geyer, 2011), also means that they each have a unique pharmacological profile. For a more detailed discussion of psychedelic drug pharmacology see Nichols and Nichols (2021).

How activation of specific serotonin receptor subtypes contributes to the psychedelic versus clinical effects in MDD is not fully understood. In clinical studies, there is a close correlation between the affinity of the psychedelic drug for 5-HT_2A_ receptors and their potency in terms of subjective effects (Egan et al., 1998; Glatfelter et al., 2022; Glennon et al., 1984; Keiser et al., 2009; López-Giménez & González-Maeso, 2017). Furthermore, the psychedelic effects of psilocybin correlate with 5-HT_2A_ receptor occupancy and plasma levels (Madsen et al., 2019) and the non-selective 5-HT_2A_/5-HT_2c_ receptor antagonist ketanserin has been shown to block the psychedelic effects of psilocybin (Kometer et al., 2013; Vollenweider et al., 1998) and LSD (Preller et al., 2017). Studies in rodent models suggest that non-psychedelic 5-HT_2A_ receptor agonists induce behavioural effects which may predict antidepressant efficacy suggesting the 5-HT_2A_ receptor is important but inducing a psychedelic effect may not be necessary (Cameron et al., 2021; Kaplan et al., 2022; Olson, 2021). Activity at other receptors is expected to occur at doses being used in clinical trials and may also contribute to efficacy (see Figure 1B). There may also be a different dose-response relationship for the antidepressant versus psychedelic effects involving distinct neural circuits (Hinchcliffe et al., 2024). Another important consideration for the pharmacology of psychedelics relates to functional selectivity or biased signalling which has been observed with the 5-HT_2A_ receptor (López-Giménez & González-Maeso, 2017). In particular, LSD binding has been suggested to induce a bias in signalling towards the β-arrestin over the canonical G_q_ signalling pathway (Wacker et al., 2017).

The activity of psychedelic drugs at serotonergic receptors is thought to induce sustained effects involving neuroplasticity and upregulation of plasticity-related genes including brain derived neurotrophic factor (BDNF), mechanistic target of rapamycin kinase (mTOR) and eukaryotic translation elongation factor 2 (EEF2) (Halberstadt et al., 2018; Vaidya et al., 1997). Part of this effect may be explained by down-stream signalling resulting from their interactions with 5-HT receptors as well as binding to intracellular 5-HT_2A_ receptors (Vargas et al., 2023). However, psilocin and LSD have also been shown to directly bind to the neurotrophic receptor tyrosine kinase 2 (trkB) receptor for BDNF (Moliner et al., 2023). These actions result in neurotrophic effects including synaptogenesis, which are thought to be important in the antidepressant effects although a direct link between these mechanisms and antidepressant efficacy has not been established in humans or translational rodent models.

How the receptor-level actions of psychedelic drugs link to their regional and network-level effects on the brain is also unclear. Psychedelic drugs may have greater effects on 5-HT_2A_ receptor-rich regions of the brain, such as the lateral/medial prefrontal cortex, anterior cingulate cortex and medial temporal lobe (Vollenweider, 1997). Individually, several of these regions contribute to the default mode network (DMN), and indeed at a network level psilocybin changes DMN functional connectivity (Siegel et al., 2024). Changes to anterior hippocampal-DMN connectivity have been found to last for weeks after psilocybin treatment (Siegel et al., 2024).

### The role for preclinical studies in psychedelic research

There are a number of important questions for the future development of antidepressants based on psychedelics; animal studies will play a key role in addressing these. These include:

- understanding whether the psychedelic and antidepressant effects of psychedelics are dissociable.
- exploring the pharmacological and neurobiological mechanisms underlying treatment effects to support identification of novel drug targets.
- evaluating the efficacy and safety of psychedelics and their derivatives including non-psychedelic 5-HT_2A_ receptor agonists.

**Figure 2:**
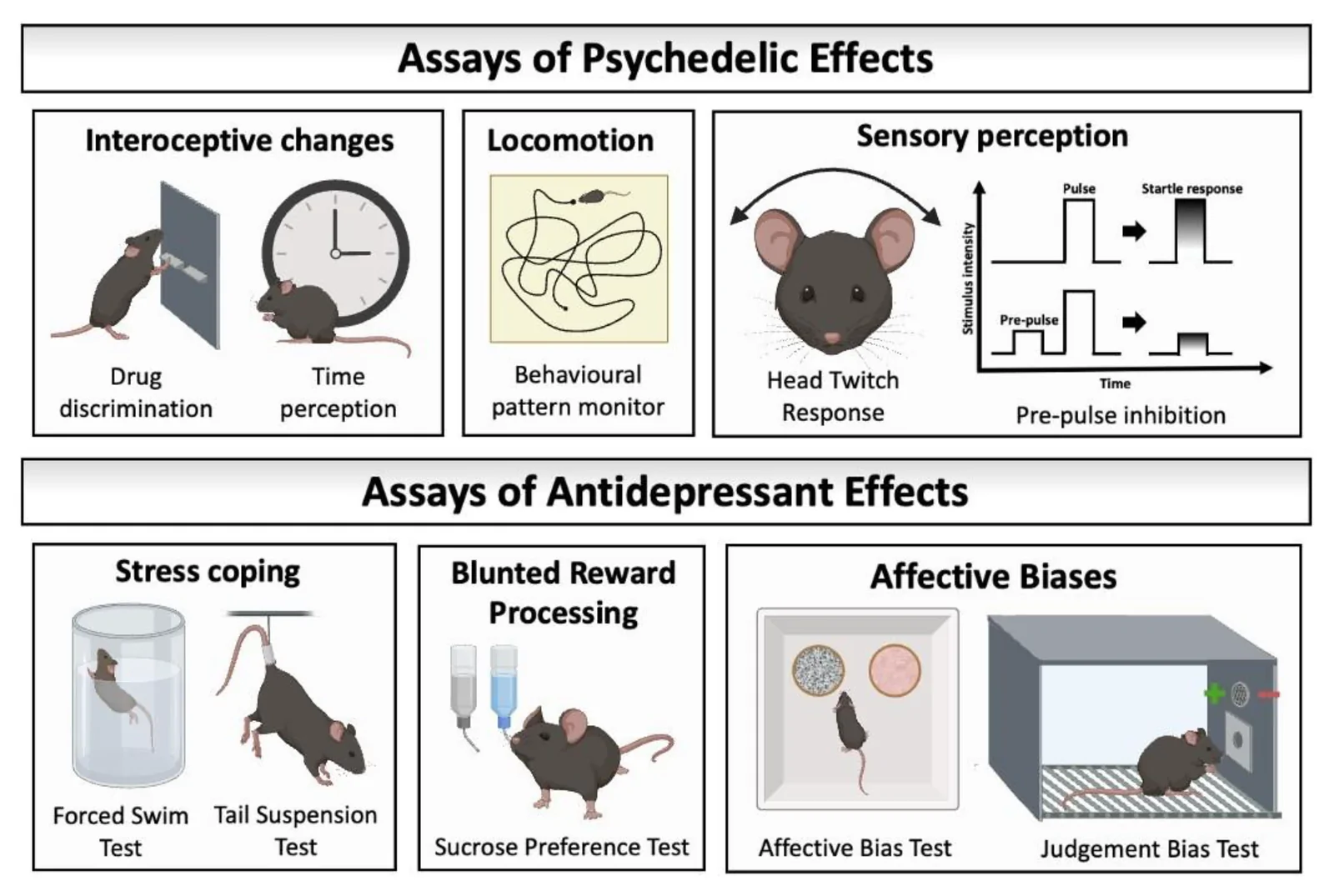
Rodent assays assessing psychedelic effects and antidepressant effects of psychedelics. Created in BioRender. Anderson, D. (2024) BioRender.com/q82g113

To achieve these objectives requires two main types of behavioural assay, one to enable predictions about antidepressant efficacy and the other hallucinatory effects (Figure 2). These may also be used in combination with a disease model e.g. chronic stress model of depression. Animal behavioural assays quantify specific behaviours thought to be analogous to specific psychological/psychiatric phenomena in humans (Becker et al., 2021). Because human studies largely depend on subjective self-report measures to understand the experience of psychedelics and its clinical efficacy, direct translation to animal studies is challenging. Conventional animal models of depression have been shown to have limitations (Hendrie & Pickles, 2013; Nestler & Hyman, 2010; Sewell et al., 2021; Slattery & Cryan, 2012) which are discussed in more detail in the following sections. Traditionally, animal assays and models can be evaluated according to three core validity measures: face validity (does the behaviour in the preclinical assay ‘look like’ the behaviour or experience measured in humans?), construct validity (are the causative factors e.g. underlying biology involved in the behaviour observed in the preclinical assay the same as those underlying the behaviour or experience measured in humans?), and predictive validity (do drugs which alter behaviour in the assay, do the same in humans?) (Prut & Belzung, 2003). For psychedelic research into MDD, there are challenges meeting these criteria. First, subjective self-report measures cannot directly compare to behavioural readouts in rodents, making it difficult for animal assays to meet face validity. In addition, there is a lack of biomarkers and a clear understanding of causal mechanisms in MDD, rendering it difficult to establish construct validity. Even in rodent models such as chronic stress models, where there is arguably a greater degree of construct and face validity (Billings et al., 1983; Horesh & Iancu, 2010; Sanacora et al., 2022), establishing the involvement of clinically relevant underlying neurobiology remains challenging due to the heterogeneous nature of depression and the lack of definitive biomarkers or a comprehensive understanding of all causal mechanisms in MDD. Finally, although behavioural assays of MDD have been validated for conventional antidepressants such as serotonin reuptake inhibitors, they have not been validated for novel antidepressants, including psychedelics, thus posing problems for predictive validity. More recently, another form of validity has been suggested: translational validity, which describes whether the output of the assay/model is measurable across humans and animals. For example, it is not possible to have a rodent model of suicidal ideation or low mood, but certain neuropsychological deficits such as affective bias can be measured across animals and humans (Robinson, 2018).

### Assays exploring the psychedelic effects in rodents

Given it is impossible to recapitulate and quantify the entirety of the psychedelic experience in preclinical models, the development of animal assays of psychedelic effects have focused on aspects of the experience that may be analogous to those experienced in humans.

#### Head twitch response

The most widely used method to ascertain hallucinatory effects in rodents is quantification of the drug-induced head-twitch response (HTR) (also sometimes quantified as wet dog shakes [WDS] in rats). Psychedelic drugs elicit the HTR (Corne & Pickering, 1967) and the effects are dose-dependent and can be blocked by 5-HT_2A_ receptor antagonists (Canal & Morgan, 2012; Vollenweider et al., 1998). There is a good correlation between the ability of psychedelic drugs to elicit a HTR in rodents and their reported psychedelic potency in humans (Corne & Pickering, 1967; Halberstadt et al., 2020). The HTR has been termed the “behavioural signature of psychedelic drugs upon stimulation of the… 5-HT_2A_ receptor in rodents” (De La Fuente Revenga et al., 2021). However, several different classes of drug elicit, potentiate or inhibit the HTR but not all of these induce or modulate the psychedelic experiences in humans (Table 1). The mechanisms which underlie the HTR/WDS are not known and other drugs which have hallucinatory effects in humans do not induce these behaviours in rodents including ketamine. The time course of effects is also relatively short compared to their effects in humans and higher doses tend to induce reduced effects consistent with an inverted U-shaped dose-response curve.

#### Pre-pulse inhibition

Pre-pulse inhibition (PPI) of the acoustic startle response is another method linked to sensory disturbances induced by psychedelics. PPI describes the inhibition of the startle response to an intense auditory stimulus by a preceding weak stimulus (Graham & Murray, 1977; Hoffman & Searle, 1968). Successful PPI reflects effective filtering of relevant sensory information to alter motor responses, termed sensorimotor gating; PPI disruption is suggested to reflect sensory ‘flooding’. PPI studies in rodents have been widely used in the evaluation of antipsychotic medications and drugs which modulate mesolimbic dopamine can attenuate PPI deficits (Johansson et al., 1995; Mansbach et al., 1988; Zhang et al., 2000). In rodents, various psychedelic drugs have been shown to disrupt PPI (Johansson et al., 1995; Padich et al., 1996; Sipes & Geyer, 1994; Yamamoto & Ueki, 1975) and this effect can be blocked by ketanserin (Padich et al., 1996). Psilocybin seems to have less reliable effects on PPI compared to other psychedelics, sometimes facilitating and sometimes inhibiting PPI (Braff et al., 2001; Geyer et al., 2001). This may be because of its action at both 5-HT_1A_ and 5-HT_2A_ receptor, with agonism at the former acting to increase PPI.

#### Drug discrimination

Drug discrimination relies on animals learning to make a specific behavioural response e.g. pressing one of two available levers, when they have experienced the drug versus when they receive a control treatment. Once the animal has learned to discriminate between the drug-paired and control-paired stimulus, a test compound can be given and the animal’s response used to determine if it will substitute for the training drug (Swedberg & Giarola, 2015). This method assumes that animals experience a specific interoceptive state induced by a drug and can learn to associate this with making a specific behavioural response; if the animal learns the discrimination then the interpretation is that the drug has generated a distinct interoceptive state from the control condition (Hirschhorn and Winter (1971). Rodents can discriminate between psychedelic hallucinogens versus dissociative or deliriant hallucinogens, stimulants, and opioids (Glennon et al., 1979; Killinger et al., 2010; Shannon, 1980; Swedberg & Järbe, 1986; Young, 2009), meaning hallucinogens with distinct subjective effects are discriminable in this paradigm. Drug generalisation (i.e. the generalisation of operant responding to one drug to other drugs of a similar class) correlates well with the underlying cellular mechanisms of drug action (Appel & Callahan, 1989) and receptor blocking studies have shown that antagonists’ ability to block both LSD discrimination and generalisation to 1-(2,5-dimethoxy-4-methylphenyl)propan-2-amine (DOM) is closely correlated with antagonist affinity to 5-HT_2A_ receptors (Winter et al., 1999).

#### Time perception

Animals treated with psychedelics also show behaviours suggestive of altered time perception. Rodents readily learn tasks which involve timing their behavioural responses to a specific schedule to achieve a food reward. Drugs which disrupt timing behaviours lead to more errors in the task. Administration of the 5-HT_2A_ receptor agonist DOI provokes earlier lever switching in a peak interval timing paradigm (where responses to one lever are rewarded in the first half of the trial and responses to another are rewarded in the latter half), suggesting that rodents were overestimating the length of the elapsed period; this effect was prevented with ketanserin (Body et al., 2003). In time discrimination tasks (where rodents must respond depending on the perceived length of a stimulus), the administration of DOI caused rodents to underestimate stimulus duration, and the effect was antagonised by the selective 5HT_2A_ receptor antagonist volinanserin as well as ketanserin (Asgari et al., 2006; Halberstadt et al., 2016; Hampson et al., 2010). Time underestimation was also seen following administration of psilocybin, and its active metabolite psilocin (Popik et al., 2022).

#### Locomotor activity

Similar to PPI assays, locomotor activity has been widely used for investigating antipsychotics. Psychomotor stimulants such as amphetamine and the NMDA receptor antagonists such as PCP increase locomotor activity in both mice and rats and these effects can be attenuated by D2 and/or 5-HT_2A_ receptor antagonists (Millan et al., 1999; Moser et al., 1995; Rebec & Bashore, 1984; Rolinski & Scheel-Krüger, 1973). Locomotor changes have also been observed following psychedelic drug administration. In early studies, Cohen & Wakeley observed a decrease in locomotion following LSD (Cohen & Wakeley, 1968), but contrasting results have been observed depending on the specific apparatus used (Dandiya et al., 1969). Mark Geyer and colleagues have tested several psychedelic drugs on the ‘Behavioural Pattern Monitor’ – an apparatus used to assess quantitative and qualitative changes in locomotion and exploration (Geyer, 1990). LSD and DOI decrease locomotor activity, exploratory behaviour, and the tendency to follow a previously explored path (Adams & Geyer, 1982), and these effects can be blocked with pre-treatment with the 5-HT_2A_/5-HT_2C_ receptor antagonist ritanserin (Mittman & Geyer, 1991) and volinanserin (Krebs-Thomson, 1998). Changes in locomotor activity are a relatively non-specific behavioural readout which can be difficult to interpret with regard to psychedelics based on current results. One of the challenges with locomotor activity studies is that the behaviour is sensitive to the specific equipment being used and environment which can lead to differences in both control behaviour and interactions with drug treatments.

#### Future strategies

Although psychedelics exhibit behavioural effects in rodents which can be interpreted as being related to hallucinatory effects in humans, better translational methods are needed. One potential translational measure which could be useful for understanding the hallucinatory effects of psychedelics in both humans and rodents relates to their effects on vision. Rodents have been trained in a variety of different cognitive tasks based on visual cues with touchscreen-based tasks and equipment widely used in mice and rats (Bussey et al., 2008). A measure of visual illusory percepts (such as illusions of motion in otherwise stationary objects or surfaces (Carter et al., 2004) may be used in different species to achieve translational validity. In macaques for example, there are reports of invisible ‘fly catching’ behaviour following intake of high doses of psilocybin, which may represent hallucinations or illusory motion (Fantegrossi et al., 2004). Visual discrimination paradigms have been performed in rats where animals are trained to discriminate between stationary and dynamic visual stimuli (vertical gratings) (Vejmola et al., 2022). In this paradigm, psilocin was shown to impair task performance. In a human version of this task where individuals were presented with static or dynamic images (faces or white ovals) and had to state whether the stimulus was moving, psilocybin similarly impaired performance. Impairment correlated with reported intensity of subjective effects, indicating the strong translational validity of this task.

Another possible approach involves measuring auditory hallucinatory phenomena in both animals and humans. This approach was taken by Schmack and colleagues who defined hallucinations as false perceptions that are experienced with equal subjective certainty as true perceptions (Schmack et al., 2021). In a psychometric task, mice were played a tone against a white noise background and trained to report their perception of the tone via an instrumental response. Their confidence was gauged from the length of time they waited for a reward following a correct response. Cases where subjects falsely reported perception of a tone with high confidence were deemed hallucination-like perceptions (HALIPs). In a human equivalent to this task, task performance positively correlated with self-reported hallucination proneness in a psychiatric questionnaire and in rodents, high-confidence false alarms in mice increased following acute administration of ketamine.

#### Summary

Several rodent assays have been used to explore different aspects of the acute psychedelic state in humans. Of those described, the HTR and drug discrimination are perhaps the most frequently used and have been most comprehensively validated. Each model has limitations, with inconsistencies between studies and a lack of specificity. Some of these inconsistencies may be related to the distinct pharmacological profiles of the different drugs; indeed, in humans, the drugs can have different subjective effects which may be related to different receptor binding profiles. There is no ‘perfect’ model – our appraisal of the face, construct and predictive validity of each is shown in Table 2. There is a central issue of translational validity as ultimately hallucinatory percepts are very difficult to assess in rodent models; conversely, the behaviours assessed in animal models are not observed in humans. Combining approaches – e.g. a model of the sensory aspects of hallucinogenesis combined with one assessing locomotion – may be most valuable.

### Assays exploring the antidepressant-like effects of psychedelics in rodents

The measurement of antidepressant-like effects of psychedelic drugs in rodents employs the use of both assays and models which have been used for decades when studying typical antidepressants. Broadly, these assays and models have been used to explore (i) stress coping (ii) blunted reward processing and, (iii) affective biases.

#### Stress coping

The forced swim test (FST) (Porsolt, Bertin, et al., 1977; Porsolt, Le Pichon, et al., 1977) and tail suspension test (TST) (Steru et al., 1985) are the two most widely used rodent behavioural assays and pharmacological screens for predicting antidepressant efficacy. In the FST an animal is placed in a container of water from which it cannot escape and the time taken for the animal to become immobile is measured. In the TST (not suitable for rats), a mouse is suspended by its tail and, similarly, the time taken to immobility is assessed. Changes in immobility time and escape behaviours involving swimming/struggling versus climbing are used as measures of antidepressant efficacy with conventional antidepressants acting to reduce immobility time (Slattery & Cryan, 2012). Inconsistent effects have been observed across different psychedelic drugs in these tests (Table 3). Explanations for this lack of consistency include the possibility of different effects across different strains of rodents, measurements being taken at different timepoints, and different effects depending on rodents’ prior experience with the assay. Thse dose range tested may also be an important factor.

The FST and TST have been criticised as having poor translational validity. These assays were originally developed as pharmacological screens for assessing the potential efficacy of monoaminergic antidepressants rather than as models of depression. Most drugs which are antidepressant in humans reduce immobility time in the FST and TST, but these tests may be less useful when assessing the therapeutic potential of RAAD (Viktorov et al., 2022). A major criticism of these tests is that behavioural effects of conventional antidepressants (e.g. SSRIs) occur very rapidly (within minutes to hours), which contrasts with the delayed therapeutic effects of these drugs in humans. Rather than reflecting a depressive phenotype or an anthropomorphic notion of ‘hopelessness’, immobility has been suggested to reflect an adaptive response toward an inescapable stressor that conserves energy to promote survival (Molendijk & de Kloet, 2015; Nadeau et al., 2022), and increased motion may reflect escape behaviours driven by elevated levels of anxiety (Anyan & Amir, 2017). In addition, as these tests rely heavily on locomotion, they are susceptible to confounding by off-target stimulant or depressant effects of drugs (Slattery & Cryan, 2012; Unal & Canbeyli, 2019). For this reason, it is pertinent to include a general test of locomotion as a control, though this is not always done, raising the possibility of yielding false positive or false negative results. Interestingly, both volinanserin and ketanserin have been shown to reduce general locomotion (Cameron, Patel, et al., 2023; Canal et al., 2013), complicating conclusions drawn from antagonist studies of psychedelics examining the role of the 5-HT_2A_ receptor in immobility behaviour. Confounds associated with motor effects are most relevant when investigating the acute effects of a drug treatment and many studies using the FST and TST to investigate RAAD test the animals 24 hours or more post-treatment when this is less relevant, although sustained effects on locomotor activity may still be present.

The ‘learned helplessness’ model is another approach for measuring stress coping and behavioural responses to adversity. Animals exposed to inescapable shocks develop passive coping strategies which have been hypothesised to reflect the human experience of ‘hopelessness’ (Seligman, 1975). This test has shown sensitivity to conventional antidepressant medications, however, psilocybin and its metabolite psilocin have variable effects on shock avoidance behaviour, reducing escape failures in one study (Shao et al., 2021) but not another (Kaplan et al., 2022). Like the FST and TST, the learned helplessness model has been criticised as a depression readout. Escape behaviours evoked by repeated foot-shocks are likely energetically costly and escape responses may be suppressed to conserve energy resources (Minor & Hunter, 2002). Furthermore, like tests of behavioural despair, shock avoidance paradigms are influenced by locomotor activity as well as nociceptive effects of drugs, and have issues with construct validity (Cryan & Mombereau, 2004).

In summary, the FST, TST and learned helplessness model have been extensively used to assess antidepressant efficacy, but their translational validity to depression is debatable. Originally designed for screening monoaminergic antidepressants, these assays may not capture the effects of psychedelics. In line with this, in a recent systematic review examining studies evaluating NMDA receptor antagonists in the FST or TST, it was observed that immobility time in these tasks did not emerge as a reliable predictor of clinical efficacy in subsequent clinical trials (Viktorov et al., 2022).

#### Blunted reward processing

Blunted reward processing is thought to be relevant to anhedonia, a core DSM-V symptom of depression (APA, 2013), which is defined as “lack of enjoyment from, engagement in, or energy for life’s experiences; deficits in the capacity to feel pleasure and take interest in things”. Typically, reward is described as comprising three elements which are believed to have distinct underlying neurobiological mechanisms: reward liking (pleasure experienced from rewards), reward wanting (motivation toward obtaining rewards), and reward learning (guiding behaviour based on previous experiences) (Berridge & Robinson, 2003).

The sucrose preference test (SPT) is the most frequently used rodent assay of reward-related behaviour, providing a measure of reward sensitivity. The SPT is a consumption test that assesses preference for a bottle containing sucrose (1-2%) over a bottle containing plain water (Willner et al., 1987). In studies of conventional antidepressants, a reduced sucrose preference is first elicited using a depression model (most often chronic stress) and rescue of this phenotype is taken as indicative of an antidepressant effect. Unlike the FST and TST, greater predictive validity is conferred to the SPT given that, similar to the therapeutic effects of conventional antidepressants in humans, changes in sucrose preference usually take several days to weeks to manifest. Psychedelics have shown variable effects on reward processing in the sucrose preference test (Table 3). In some studies, this may be related to the failure to first establish an anhedonic phenotype. For instance, the chronic restraint stress model used by De Gregorio et al. (2022) failed to elicit a deficit in sucrose preference, while other studies were conducted in naïve animals. Even where sucrose preference deficits are successfully elicited, these are not always consistent. For instance, Hesselgrave et al. (2021) found that a subset of animals were not stress-susceptible. Like the FST and TST, the SPT was originally developed for conventional monoaminergic antidepressants and stress-induced impairments in reward sensitivity. However, this once again raises questions about the effectiveness of this test to evaluate the therapeutic mechanisms of a distinct category of drugs, such as psychedelics. Translatability of the SPT is limited and in an equivalent test for humans, the sweet taste test, MDD patients are not different to controls (Dichter et al., 2010). In fact, it has been demonstrated that consumption of palatable foods actually increases in response to chronic stress in humans (Torres & Nowson, 2007).

Rather than measuring food-related appetitive behaviour, the female urine sniffing test (FUST) assesses reward sensitivity by measuring appetitive responses of male rodents to female urine (Malkesman, 2011). Male mice display a preference for female urine in oestrus, compared to male urine and 10-14 days of chronic mild stress can reduce this preference, parallelling experiences of diminished interest or pleasure in typically rewarding activities in MDD. Psilocybin reverses this reduction in preference (Hesselgrave et al., 2021), in line with clinical evidence that psilocybin treatment significantly improves sexual dysfunction up to 6 months after treatment in people with depression (Carhart-Harris et al., 2018). The FUST could be described as a translational measure of impairments in reward as it measures behaviour instinctively exhibited by rodents. However, it is important to note that assessing just one variable (i.e. time spent sniffing) may not capture the full spectrum of anhedonic symptoms experienced by patients with MDD, particularly those that are not related to sexual cues.

A more direct method of assessing reward sensitivity is achieved by measuring intracranial self-stimulation (ICSS) (Olds & Milner, 1954). Here, rats self-administer electrical brain stimulation via microelectrodes in the mesolimbic dopamine system. In one study, acute LSD administration was not shown to affect current-intensity thresholds, suggesting no acute effects on reward sensitivity (Elsilä et al., 2022). However, using the frequency-rate paradigm, repeated LSD treatment was shown to reverse attenuation of ICSS depression in rats induced by the pro-depressant kappa opioid agonist U69,593 (Sakloth et al., 2019). Although ICSS has been well-validated for its ability to predict the abuse potential of drugs, its utility in predicting drug efficacy for depression treatment is not as well-validated.

Tasks such as the progressive ratio task (PRT) and effort-based tasks provide methods for measuring the motivational effects of psychedelics. In the PRT, animals are presented with a consecutive series of trials and, on each trial, they must exert progressively more effort (more lever presses) to obtain the same food reward. This provides a way of assessing the maximal effort that an individual will exert for a reward (‘breakpoint’). In this paradigm, acute low doses of psilocybin (0.05-0.1 mg/kg) increased breakpoints in rats with low baseline performance (Higgins et al., 2021). However, no effect was found with a larger 1 mg/kg dose of psilocybin (Roberts et al., 2023). This task shows some translational validity in that lower breakpoints are found in unipolar and bipolar individuals compared to controls (Hershenberg et al., 2016). However, rodent models of depression, such as chronic stress and maternal separation, do not produce consistent effects on motivation in this task (Amitai et al., 2019; Barr & Phillips, 1998; Shalev & Kafkafi, 2002). Furthermore, performance may be confounded by acute, drug-induced motor impairments (Slaney et al., 2018). Studies investigating the effects of psychedelics often test animals at time points after the drug has cleared, e.g. 24 hours post-treatment, to investigate any sustained effects on motivation when acute effects on locomotor activity will have less impact, although there still remains the possibility that locomotor effects may persist. With these limitations in mind, effort-based decision-making tasks, such as the Effort for Reward Task (EfR), have been developed where an animal is required to decide between a high effort/high value reward and a low effort/low value reward (Griesius et al., 2020; Marangoni et al., 2023). Reverse translation of this task into a human equivalent indicates its translational value, however as psychedelics are yet to be tested in this task, the EfR task remains an important assay for future studies.

With the development of objective computer-based psychological tasks in human depression studies, another recent development is the translation of human reward tasks into rodent paradigms for quantifying reward learning in rodents. Examples of this are the Response Bias Probabilistic Reward Task (RBPRT) (Der-Avakian et al., 2013; Der-Avakian et al., 2017; Piantadosi et al., 2016) and the Probabilistic Reversal Learning Task (PRLT) (Bari et al., 2010). Both tasks assess the ability of individuals to learn and adapt their behaviour based on probabilistic feedback. Subjects are presented with two or more stimuli and are required to choose between them. Each stimulus is associated with a probabilistic outcome of reward which, in the RBPRT, varies over time and, in the PRLT, is intermittently reversed. Depressed populations exhibit impairments in human versions of both tasks (Mukherjee et al., 2020; Pizzagalli et al., 2008). In rodents, paradigms using operant chambers with spatial cues, tones or touchscreens have been developed, as well as more ethologically valid bowl-digging paradigms (Griesius et al., 2023; Jackson et al., 2024). Although the effect of psychedelics on reward learning in these tasks remains the subject of ongoing investigation, ketamine has been tested, though with conflicting results. Whereas Rychlik et al. (2017) found that ketamine reduced sensitivity to misleading negative feedback in the PRLT, Wilkinson et al. (2020) found that ketamine, at higher doses, impaired reward learning, positive feedback sensitivity, and general task performance.

A related rodent assay of reward learning is the Reward Learning Assay (RLA) developed by the author’s laboratory. Here, rats learn to discriminate between two cues e.g. digging substrates, one containing a food reward and one without. During independent learning sessions, the rewarded cue is associated with either a low or high value reward. When a choice test is subsequently carried out, healthy control rats exhibit a reward-induced bias towards the substrate associated with high value reward. This is impaired in depression models, similar to the reward learning deficits in depressed patients described above. In fact, unlike the SPT which is only sensitive to chronic stress, the RLA shows remarkable consistency in sensitivity across different depression models for reward learning impairments (Robinson, 2018 plus Stuart et al., 2017, 2019). A similar paradigm has shown preliminary success in mice (Graulich et al., 2016). Recent unpublished work from our laboratory found that, 24 hours after psilocybin treatment, the reward learning deficit exhibited by rats chronically treated with an inflammatory agent, IFN-α (interferon-α), was reversed and rats were also able to develop a high value choice bias in a subsequent RLA, indicating that psilocybin may rapidly amelioriate reward learning impairments (Hinchcliffe et al., 2022).

With the transition from subjective, questionnaire-based approaches to the development of novel computer-based tasks in human studies of depression, reverse translation of human tasks provides novel, simple and translationally valid approaches for studying the effect of psychedelics on reward processing, such as the EfR, PRLT and RBPRT. However, it is important to note that each of these tests only assess one component of reward. These tests may therefore be most useful when combined in a battery of tasks probing different reward components, allowing one to determine the specific aspects of reward affected by psychedelics. In depressed patients, psilocybin has been shown to improve symptoms associated with deficits in reward processing (Carhart-Harris et al., 2016). This core symptom is often resistant to conventional antidepressant treatment and animal studies also suggest improvements in reward processing with RAADs including psychedelics. However, it should be noted that in rodents the SPT and FUST also respond to chronic SSRI treatment.

#### Affective biases

Another approach to developing translational assays for investigating the mechanisms of depression in rodents are based on objectively defined neuropsychological impairments seen in patients. Depressed individuals exhibit negative affective processing biases in domains of learning, memory, attention, and emotional interpretation (Erickson et al., 2005; Kyte et al., 2005; Murphy et al., 2003; Persad & Polivy, 1993; Rubinow & Post, 1992), and recent evidence indicates these may be ameliorated by psychedelic drugs. For example, both LSD and psilocybin reduce recognition of negative emotional expressions (Barrett et al., 2020; Dolder et al., 2016; Kometer et al., 2013), while psilocybin biases behaviour toward positive emotional cues in an attention task (Kometer et al., 2013). Two types of rodent task have been developed, the Affective Bias Test (ABT) which quantifies affective bias associated with reward learning and memory and judgement bias/ambiguous cue interpretation tasks (Judgement Bias Task, JBT) which quantify affective biases associated with decision-making behaviour.

The ABT is a reward-based associative learning task that measures affective biases in learning and memory in rats. Animals learn to associate a specific cue, a digging substrate, with finding a food reward. By keeping the value of the reward constant and asking animals to choose between the experience they encountered during the affective manipulation versus a control condition, affective state-induced memory biases can be quantified. An animal in a positive affective state exhibits a positive bias towards the cue learnt during the manipulation whereas an animal in a negative affective state shows a negative bias. Extensive validation confirms sensitivity to positive and negative affective state manipulations generated using either pharmacological or psychosocial manipulations (Hales et al., 2023; Hinchcliffe et al., 2017; Refsgaard et al., 2016; Stuart et al., 2013, 2015; Stuart et al., 2017). However, it is important to note that a mouse version of this task was not found to be sensitive to a positive affective manipulation (social enrichment) (Graulich et al., 2016). In line with evidence that acute SSRI treatment can positively bias emotional processing in humans (Harmer et al., 2009), conventional antidepressants have been shown to positively bias learning of new substrate-reward associations in the ABT (Stuart et al., 2013). In contrast, two pharmacologically distinct RAADs, ketamine and scopolamine, attenuate the retrieval of a negative bias associated with a past experience when given shortly before the choice test (Stuart et al., 2013, 2015). When animals are tested 24 hours after an acute dose of ketamine, the negative bias is reversed, with animals exhibiting a positive bias consistent with re-learning (Hinchcliffe et al., 2024). These effects on past experiences are not seen with conventional antidepressants and are hypothesised to provide an explanation for the temporal differences in the time course of clinical effects observed in patients (Hinchcliffe et al., 2024; Stuart et al., 2015). Recent evidence from our lab shows that psilocybin both induces positive affective biases during the learning of new reward associations and remediates negative biases in the ABT both acutely and after 24 hours, thus seeming to bridge the effects of conventional ADs and novel RAADs (Hinchcliffe et al., 2024).

In the JBT, ketamine also exhibits a very different time course of effects when compared to conventional antidepressants (Hales et al., 2017). This task assesses whether the affective state of an animal biases decision-making in response to an ambiguous stimulus. Animals are trained to respond to two distinct cues with different outcomes (e.g. high value or low value reward). Animals are then presented with a novel ambiguous cue and their responses, indicating anticipation of high value or low value reward, are measured. In this task, ketamine was shown to induce more ‘optimistic’ behaviour following an acute dose while the conventional antidepressants only achieved this effect after chronic administration (Hales et al., 2017). Like the ABT, the distinct effects of conventional antidepressants and RAADs in the JBT are intriguing and may enhance the predictive value of this task. However, psychedelics are yet to be assessed in this paradigm.

It is important to note that these behavioural methods are more technically challenging than conventional assays, requiring specialist equipment and/or expertise. This will inevitably limit throughput and thus their more general use in preclinical research and drug development, but it may be possible to use these methods to establish new assays based on automated behavioural analysis. Tasks such as the ABT provide an objective measure of affective state which can then be used to validate less specialised measures, such as 50KHz ultrasonic vocalisations which are directly related to affective biases and can be automatically quantified using non-specialist equipment and freely available AI technology (Hinchcliffe et al., 2020).

The ABT and JBT offer a translational approach to assessing the antidepressant effects of psychedelics given they are based on observable neuropsychological impairments in depression and their demonstrated sensitivity to both positive and negative affective state manipulations in rodents (Hinchcliffe et al., 2017; Refsgaard et al., 2016; Stuart et al., 2013, 2015; Stuart et al., 2017). Interestingly, in both the ABT and JBT, the effects of ketamine were localised to the medial prefrontal cortex (mPFC) (Hales et al., 2020; Stuart et al., 2015), a region associated with emotional processing (Etkin et al., 2011) and known to be affected in depression (Campbell et al., 2004). DOI infusions into the mPFC also modulate affective biases (Hinchcliffe et al., 2024). This suggests some degree of construct validity and hints at the potential value of such tasks for unpacking the neurobiological mechanisms underlying the antidepressant effects of psychedelics in future studies.

#### Summary

Animal studies using traditional assays for screening antidepressants such as the FST, TST, SPT have found inconsistent results in assessing the antidepressant effects of psychedelics (Table 4). One possible reason for this is that these variations are due to differences among different psychedelics (e.g. psilocybin vs LSD vs DMT), but initial clinical findings would suggest that they are all effective RAADs. These assays were designed and validated based on conventional, delayed-onset antidepressants and may be limited in sensitivity to drugs with a different pharmacology (Berton & Nestler, 2006; Robinson, 2016; Trunnell & Carvalho, 2021). Evaluating the face validity of these tasks encounters challenges due to anthropomorphism, and construct validity is hindered by our current limited understanding of the causal mechanisms underlying MDD and a lack of reliable biomarkers. For example, though chronic stress protocols induce deficits in most of the assays described above, their sensitivity to other precipitating factors such as early life adversity and inflammatory models of MDD is less clear (Deak et al., 2005; Dimatelis et al., 2016; Kentner et al., 2010; Savignac et al., 2011; Wang et al., 2011). One argument used to support the ongoing use of these assays is that they are the best available paradigms; however, we have discussed alternative approaches which offer greater translational validity and have been developed based on the neuropsychological impairments which are seen in MDD patients. The recent developments in methods to study affective biases and reward learning impairments in rodents may provide more appropriate models to study the antidepressant effects of psychedelics and involve a relevant underlying neuropsychological mechanism (Table 4). However, further innovation and careful validation of behavioural readouts for psychedelic research are still needed.

### Outstanding questions and challenges

#### Psychedelic vs antidepressant effects

One of the biggest challenges in animal studies of psychedelics is the limited translational validity of many currently available assays of psychedelic and antidepressant effects. However, with the move toward developing objective measures in humans, we have seen ongoing progress focused on translating such measures into rodent assays.

One of the important purposes of animal studies is to understand underlying mechanisms so better treatments can be achieved. Debate exists over whether the subjective, psychedelic effects of psychedelics in humans are necessary for their therapeutic benefits. Human studies show that there is a correlation between the subjective experience and antidepressant efficacy, suggesting interdependent underlying mechanisms (Griffiths et al., 2016; Ross et al., 2016). However, these subjective effects limit the scalability of psychedelics as treatment due to the need for extended periods of psychological support, making them unsuitable for many clinical settings and some patients. Preclinical studies have contributed to this debate by assessing whether psychedelics effects in rodent assays of antidepressants are dependent on 5-HT_2A_ receptor activation, given that psychedelic effects in humans seem to rely on 5-HT_2A_ receptor activation. Studies in rodent models suggest 5-HT_2A_ receptor agonism alone is not sufficient for an antidepressant response and blocking 5-HT_2A_/5-HT_2C_ receptors does not prevent the antidepressant-like effects of psilocybin (Cameron, Benetatos, et al., 2023). For example, ketanserin had no effect on psilocybin effects in the SPT or FUST (Hesselgrave et al., 2021), however the ketanserin dose used (1 mg/kg) blocks only around a third of 5-HT_2A_ receptors (Smith et al., 1995). A higher dose (4 mg/kg) completely blocked the effect of 5-MeO-DMT on FST immobility (Cameron, Patel, et al., 2023), but the selectivity of ketanserin for 5-HT_2A_ receptors is limited at higher concentrations (Casey et al., 2022). In another study, the more selective 5-HT_2A_ receptor antagonist volinanserin similarly blocked psilocybin’s effect on FST immobility (Takaba et al., 2023), however, these findings may be confounded by the fact that volinanserin has its own effects on immobility time in the TST (Kaplan et al., 2022). Other studies have used 5-HT_2A_ receptor knock-out mice lines but have found conflicting effects of psilocybin on FST and SPT behaviour in 5-HT_2A_-/- mice, possibly due to differences in the strain and stress model used (Cameron, Patel, et al., 2023; Sekssaoui et al., 2024).

#### Non-psychedelic analogues

Recently, and relatedly, there has been growing interest in non-psychedelic compounds targeting 5-HT_2A_ receptors for potential clinical use in humans. Some 5-HT_2A_ receptor agonists like lisuride, ergotamine and 2-Bromo-LSD are said to be non-psychedelic in humans (López-Giménez & González-Maeso, 2018), possibly due to biased agonism at the receptor (González-Maeso et al., 2007). Analogues of some psychedelic compounds e.g. 2-Bromo-LSD, tabernanthalog (TBG, structurally related to ibogaine), (R)-70 and AAZ-A-154, have been shown to reduce immobility in the FST or ameliorate deficits in the SPT while inducing few or no HTRs (Cameron et al., 2021; Dong et al., 2021; Kaplan et al., 2022; Lewis et al., 2023). Such studies have been taken to suggest that these compounds retain antidepressants properties whilst being non-psychedelic. This assumes the absence of HTRs implies non-psychedelic potential. Most of these compounds have not been tested in humans, so their lack of psychedelic effects remains unconfirmed. Of those that have reported side effects of lisuride and ergotamine include hallucinations and delusions, particularly at higher doses (Gulbranson et al., 2012; Lees & Bannister, 1981; Neophytides et al., 1982; Vaamonde et al., 1991), and 2-Bromo-LSD has been reported to induce some minor psychoactive effects (Karst et al., 2010). Furthermore, as we have seen, the behavioural assays in which these putative non-psychedelic analogues have been tested may not accurately predict antidepressant efficacy in humans and clinical trials remain to be carried out.

#### Non-5HT_2A_ mechanisms of antidepressant effects

Furthermore, the relative contributions of different serotonergic and non-serotonergic receptors to psychedelic drugs’ antidepressant effects remains unclear. Most serotonergic psychedelics also have potent 5-HT_2B_ and 5-HT_2C_ binding (Nelson et al., 1999), and binding to trkB receptors within clinically relevant dose ranges (Moliner et al., 2023). Some psychedelics, such as DMT, have higher affinity for the 5-HT_1A_ receptor whereas others (phenylethylamines) do not, but give a positive antidepressant signal in conventional rodent models (Cameron, Benetatos, et al., 2023). There is also evidence from animal studies that the effects of the non-psychedelic analogues lisuride and TBG in rodent depression assays are lost in 5-HT_2A_-/- knockout mice whereas the effects of DOI and psilocybin are preserved (Sekssaoui et al., 2024). In addition, neither 5-HT_1A_, D_1_ or D_2_ receptor blockade altered the effects of psilocybin. This suggests that psychedelic drugs (and non-psychedelic 5-HT_2A_ receptor agonists) may induce rapid antidepressant effects through both 5-HT_2A_-dependent and 5-HT_2A_-independent mechanisms. However, the interpretation of these findings depends on the validity of the behavioural readout and may not translate to the findings in humans.

#### Sex differences

There are other challenges to applying the findings of preclinical psychedelic research to humans. Most preclinical studies are conducted exclusively in males, however sex-differences in performance are evident in various animal assays of psychedelic effects, including the HTR (Dunlap et al., 2020; Jaster et al., 2022), drug-discrimination (Herr, 2017), and PPI (Vohra et al., 2022) (Tylš et al., 2016). Furthermore, female rodents’ behaviour in assays of psychedelic effects differs according to their oestrus cycle (Páleníček et al., 2010) (Tylš et al., 2016). Sex-differences have also been noted in depression assays, such as the FST, learned helplessness model, and SPT (Dalla, 2008; Drossopoulou et al., 2004; Pitychoutis et al., 2009). Beyond behavioural assays, neuroplastic changes induced by psychedelics also vary by sex (Cameron et al., 2019; Shao et al., 2021). Incorporating sex as a factor in future studies will be important in future preclinical assessments of psychedelic substances.

#### Dose selection

A final important consideration is the need for animal studies to use appropriate psychedelic doses and that these achieve clinically relevant receptor occupancy. For most drugs, there is a narrow range of doses that are both clinically effective and well-tolerated. However, dose selection in preclinical research often lacks justification, and this issue extends to psychedelic studies. Determining the right psychedelic dose in rodents is not straightforward as there is limited pharmacokinetic data or receptor occupancy data available in the literature. It is widely assumed that size-based dosing (i.e. mg/kg) does not account for metabolic variations or differences in the pharmacology of the receptors between species. Allometric scaling, based on body surface area, provides one method for dose conversion (Nair & Jacob, 2016). Animal equivalent doses for rats and mice calculated based on allometric scaling of doses used in clinical trials are as follows: psilocybin (mouse = 3.85 mg/kg, rat = 1.93 mg/kg), LSD (mouse = 0.03 mg/kg, rat = 0.015 mg/kg) DMT (mouse = 1.23-3.7 mg/kg, rat = 0.62-1.85 mg/kg), 5-MeO-DMT (mouse = 0.31-2.775 mg/kg, rat = 0.15-1.39 mg/kg). However, allometric scaling does not consider differences in receptor pharmacology between species. For instance, there are species differences in the 5-HT_2A_ receptor with the human 5-HT_2A_ receptor having a 15-fold higher affinity to radio-labelled psilocin relative to the rat 5-HT_2A_ receptor (Almaula et al., 1996; Gallaher et al., 1993; Johnson et al., 1994). Therefore, another approach is to find doses that produce equivalent effects on a target receptor within the brains of experimental animals as a clinical dose does in humans (Kapur et al., 2003). Achieving a 5-HT_2A_ receptor occupancy of 40-70% is important for producing psychedelic effects in humans, based on studies using psilocybin (Knudsen, 2023). Unfortunately, receptor occupancy studies in rodents are scarce in psychedelic research. Informed dose selection and comprehensive characterisation of pharmacokinetics and pharmacodynamics in rodents are essential for moving the field forward.

## Conclusions and recommendations

In this review, we have examined assays commonly used to assess the psychedelic and antidepressants effects of psychedelics in rodents and critically appraised their translational utility. Although many of the tests find effects, most are not particularly specific and, for this reason, it is unlikely that any one of these traditional tests provide an unequivocal measure of psychedelic or antidepressant effects in rodents. Many of the issues stem from the fact that psychedelic preclinical research has unique challenges. Psychedelic rodent studies try to model two constructs (psychedelic experience and depression) that are inherently subjective and linked to human experience, thus impossible to recapitulate in animals. With recent progress in developing methods for objectively measuring aspects of psychedelic effects (e.g. illusory percepts and HALIPs) and depression (e.g. reward learning deficits and affective biases) in humans, we have seen a move toward translating these types of measures into rodent assays. Greater use of these translational approaches, as well as further innovation in this area, will improve the interpretation of animal studies and facilitate more appropriate interpretation of experimental outcomes from preclinical psychedelic studies. As our understanding of the effects of psychedelics in humans evolves with the increasing numbers of clinical trials, the potential to relate these findings to animal studies will also benefit. For example, a single dose of psilocybin can induce a rapid and sustained antidepressant effect in patients which can last for many months and long after the drug has been metabolised and excreted. These findings are consistent with animal studies where both rapid and sustained antidepressant-like effects are observed after a single dose.

Ensuring appropriate doses of drugs are used is critical to engaging relevant underlying mechanisms particularly given the spectrum of receptors and diversity of pharmacology seen with psychedelics. There are also limitations associated with available receptor blocking tools, including the non-specificity of commonly used 5-HT_2A_ receptor antagonists like ketanserin, and the non-specific behavioural effects of 5-HT_2A_ receptor knock-out in mice. In addition, the impact of sex differences in the behavioural assays is still poorly understood both in terms of baseline differences and their interactions with psychedelic drug treatment. The progress of this field will depend on these limitations being addressed in future psychedelic studies in animals. Continued interdisciplinary efforts are essential for advancing methodologies which can better support research to understand the pharmacology of psychedelic substances and how these relate to their behavioural effects and antidepressant potential.

## Figures and Tables

**Table 1:** Drugs acting on a diverse array of neurotransmitter systems modulate HTRs.

System	Drug	Effect on HTR	References
**Serotonergic**	5-Hydroxytryptophan, most psychedelic hallucinogens, serotonin-releasing agents (e.g. fenfluramine), 5-HT_1A_ receptor antagonist WAY-100635	Elicits HTR	Corne et al. (1963); Corne & Pickering (1967); Darmani (1998a, 1998b)
	Monoamine oxidase inhibitors (MAOIs) (e.g. harmine, iproniazid), RS-10221	Potentiates HTR	Corne et al. (1963); Tadano et al. (2001)
	Non-hallucinogenic 5-HT_2A_R agonists (e.g. lisuride), some psychedelic hallucinogens (e.g. ALD-52), ergometrine	No effect	Corne et al. (1963); González-Maeso et al. (2007)
**Adrenergic and noradrenergic**	Yohimibine	Elicits HTR	Corne and Pickering (1967)
	Clenbuterol	Inhibits HTR	Marek and Ramos (2018)
	Ergometrine, amphetamine	No effect	Corne & Pickering (1967)
**Glutamatergic**	Certain dissociative hallucinogenics (e.g. PCP)	Elicits HTR	Corne & Pickering (1967); Nabeshima et al. (1987)
	mGlu_2/3_R agonists (e.g. eglumegad, LY379268, LY404030)	Inhibits HTR	Aghajanian and Marek (2000); Gewirtz (2000)
	Antipsychotics (e.g. haloperidol)	Inhibits HTR	Corne et al. (1963)
**GABAergic**	Benzodiazepines	Elicits HTR	Tadano et al. (2001)
	Barbiturates, nalorphine	No effect	Corne et al. (1963)
**Cholinergic**	Certain deliriants (e.g. atropine, scopolamine)	Elicits HTR	Corne & Pickering (1967)
**Histaminergic**	Antihistamines	Inhibits HTR	Corne et al. (1963)
**Opioid**	Morphine, methadone, pethidine	Inhibits HTR	Corne et al. (1963)
**Cannabinoid**	CB1 antagonists (e.g. SR-141716A)	Elicits HTR	Janoyan et al. (2002)
**Other**	orotyl-histidyl-proline amide (CG3509)	Elicits HTR	Fone et al. (1989)
	Phenytoin	Potentiates HTR	Corne et al. (1963)
	EPPTB (N-(3-ethoxyphenyl)-4- (pyrrolidin-1-yl)-3- (trifluoromethyl)benzamide)	Inhibits HTR	Shahar et al. (2022)

**Table 2:** Appraisal of the face, construct and predictive validity of animal assays assessing psychedelic effects of psychedelics.

Construct	Model	Face validity?	Construct validity?	Predictive validity?
**Changes to sensory perception**	Head twitch response (HTR)	May resemble sensory disturbances during hallucinogenesis, but peaks after 30 minutes, shorter than human psychedelic experiences.	Dependence on 5-HT_2A_ receptor, like human psychedelic experiences. May involve brain regions other than those responsible for hallucinogenesis as 5-HT_2A_ receptors are widely distributed in the brain.	Hallucinogenic potency correlates with HTRs. Most drugs lacking HTR induction are non-hallucinogenic, but some exceptions.
	Pre-pulse inhibition (PPI)	Does not resemble the psychedelic state in humans.	Dependent on the 5-HT_2A_ receptor, but also dependent on several other receptor types (e.g. D_2_/D_3_ and 5-HT_1A_ receptors), which may not be involved in psychedelic hallucinogenesis.	Hallucinogenic drugs do typically inhibit rodent PPI, however other non-hallucinogenic drug classes also inhibit PPI.
**Changes to interoceptive states**	Drug discrimination	Relies on inducing distinct interoceptive experiences which may be relevant to psychedelics but may not fully mirror hallucinogenesis.	Rodents generalise across hallucinogens with similar underlying receptor mechanisms.	Rodents generalise within hallucinogenic class and distinguish between different classes that have different effects in humans, but with occasional false positives (e.g., lisuride).
	Time perception	Alterations in time perception occur in psychedelic experiences.	5-HT_2A_ receptor involved in time perception, but alongside other receptor subclasses.	Not specific to psychedelic hallucinogens.
**Changes to motor function**	Effects on locomotor and exploratory behaviour	Locomotor effects may match part of the acute psychedelic experience.	Locomotor effects depend on both 5-HT_2A_ and 5-HT_2C_ receptors, however it is likely that different brain regions are important in locomotor vs. hallucinogenic effects of psychedelics.	Most psychedelic drugs have locomotor effects, however effects are non-specific and many non-psychedelic drug classes affect locomotion.

**Table 3:** Psychedelics have inconsistent effects in traditional rodent assays of depression.

Construct	Assay	Drug	Effect on assay	References
**Stress coping**	Forced Swim Test	Psilocybin/psilocin	Decreased immobility time	Hibicke et al. (2020); Sekssaoui et al. (2024); Takaba et al. (2023)
			Increased immobility time	Wojtas et al. (2022)
			No effect	Hesselgrave et al. (2021); Jefsen et al. (2018); Wojtas et al. (2022)
		LSD	Decreased immobility time	Hibicke et al. (2020); Cao et al. (2022)
			No effect	De Gregorio et al. (2021, 2022)
		DMT	Decreased immobility time	Cameron et al (2018; 2019)
		5-MeO-DMT	Decreased immobility time	Cameron et al (2023)
		Ayahuasca	Decreased immobility time	Lima et al. (2007); Pic-Taylor et al. (2015); da Silva et al. (2022)
	Tail Suspension Test	Psilocin	Decreased immobility time	Kaplan et al. (2022); Takaba et al. (2023)
		LSD	Decreased immobility time	Cao et al. (2022)
	Shock Avoidance	Psilocybin/psilocin	Decreased escape failures	Shao et al. (2022)
			No effect	Kaplan et al. (2022)
**Blunted reward processing**	Sucrose Preference Test	Psilocybin	Rescued stress-induced deficit in sucrose preference	Cameron et al (2023); Hesselgrave et al. (2021); Kaplan et al. (2022); Sekssaoui et al. (2024)
		LSD	No effect	De Gregorio et al. (2021, 2022)
	Progressive Ratio Task	Psilocybin	Increased breakpoints	Higgins (2021)
			No effect	Roberts (2023)
	Female Urine sniffing test	Psilocybin	Attenuated stress-induced impairments	Hesselgrave et al. (2021)

**Table 4:** Appraisal of the face, construct and predictive validity of animal assays assessing antidepressant effects of psychedelics.

Construct	Assay	Face validity?	Construct validity?	Predictive validity?
**Stress coping**	Forced Swim Test, Tail Suspension Test, Shock avoidance	Immobility resembles hopelessness but this may reflect a normal adaptive response in rodents.	Underlying mechanisms unclear. Not sensitive to risk factors for MDD such as early life adversity or immune-mediated depression.	Do not consistently predict the antidepressant efficacy of psychedelics. Susceptible to locomotor confounds.
**Blunted reward processing**	Sucrose Preference Test	Depressed patients show variable effects in comparable sweet taste tests.	Sensitive to chronic stress manipulations, but sensitivity to other affective manipulations unclear.	Predicts antidepressant efficacy of psilocybin, but not other psychedelics.
	Intracranial Self- Stimulation	Some alignment with reduced interest in rewarding activities.	Area of stimulation is part of the reward circuit believed to underlie anhedonia but task susceptible to locomotor confounds.	LSD reverses reduction in ICSS in rate frequency paradigm, but no effect on psychedelics on current-intensity thresholds.
	Progressive Ratio Task	Some alignment with reduced motivation to engage in rewarding activities.	Depressed patients impaired in this task, but inconsistent effects in different rodent depression models.	Predicts antidepressant efficacy of psilocybin at low dose.
	Reward Learning Assay	Aligns with reward learning impairments seen in MDD	Sensitive to affective state manipulations.	Psilocybin reverses impairment in reward learning.
**Affective biases**	Affective Bias Test	Aligns with objective changes in emotional processing and affective biases observed in MDD	Sensitive to positive and negative affective state manipulations and dependent on PFC, a region known to be involved in depression.	Psilocybin (and ketamine) reverses negative affective biases.

## Data Availability

N/A as this is a review.

## Bullet point summary

### What is already known

- Clinical trials show rapid and sustained antidepressant effects following acute psychedelic treatment e.g. psilocyin, DMT.
- The neurobiological mechanisms underlying the antidepressant versus psychedelic effects is unclear.

### What this study adds

- A critical assessment of the behavioural methods used in animals to investigate the effects of psychedelics.
- Discussion of their limitations and future strategies which could improve translation.

### Clinical significance

- Understanding what underlies the antidepressant effects may lead to more effective and safer treatments.

## Abbreviations

MDD: major depressive disorder
NMDA: N-methyl-D-aspartate
DMT: N,N-dimethyltryptamine
RAAD: rapid-acting antidepressant
TRD: treatment-resistant depression
SSRI: selective serotonin reuptake inhibitor
5-MeO-DMT: 5-methoxy-N,N-dimethyltryptamine
2C-B: 2,5-Dimethoxy-4-bromophenethylamine = 2C-B
25I-NBOMe: 2-(4-Iodo-2,5-dimethoxyphenyl)-N-[(2-methoxyphenyl)methyl]ethanamine = 25I-NBOMe
DOI: 1-(4-Iodo-2,5-dimethoxyphenyl)propan-2-amine
LSD: lysergic acid diethylamide
5-HT: 5-Hydroxytrytophan
CNS: central nervous system
PCP: phencyclidine
mTOR: mechanistic target of rapamycin kinase
EEF2: eukaryotic translation elongation factor 2
trkB: neurotrophic receptor tyrosine kinase 2
BDNF: brain-derived neurotrophic factor
HTR: head twitch response
WDS: wet dog shake
PPI: pre-pulse inhibition
DOM: 2,5-dimethoxy-4-methylamphetamine
HALIP: hallucination-like perception
FST: Forced Swim Test
TST: Tail Suspension Test
SPT: Sucrose Preference Test
FUST: Female Urine Sniffing Test
ICSS: intracranial self-stimulation
PRT: Progressive Ratio Task
EfR: Effort for Reward Task
RBPRT: Response Bias Probabilistic Reward Task
PRLT: Probabilistic Reversal Learning Task
RLA: Reward Learning Assay
IFN-⍰: interferon-⍰
ABT: Affective Bias Test
JBT: Judgement Bias Task
mPFC: medial prefrontal cortex
TBG: tabernanthalog
